# Prevalence and Characteristics of Eating Behavior Disorders Among Children and Adolescents

**DOI:** 10.7759/cureus.86586

**Published:** 2025-06-23

**Authors:** Islam Erraoui, Amal Hamami, Aziza Elouali, Babakhouya Abdeladim, Maria Rkain

**Affiliations:** 1 Pediatrics, University Hospital Center (CHU) Mohammed VI, Oujda, MAR; 2 Pediatrics, Faculty of Medicine and Pharmacy, Mohammed I University, Oujda, MAR; 3 Faculty of Medicine and Pharmacy, Mohammed I University, Oujda, MAR; 4 Pediatric Gastroenterology, University Hospital Center (CHU) Mohammed VI, Oujda, MAR

**Keywords:** anorexia nervosa, bulimia nervosa, children and adolescents, eating disorders, risk factors, social media

## Abstract

This descriptive cross-sectional study was conducted between January and March 2024 at the University Hospital in Oujda and several educational institutions, involving a randomly selected sample of children and adolescents aged 7-18 years. The University Hospital Center of Oujda and the selected educational institutions were chosen for their accessibility and their capacity to represent a broad spectrum of children and adolescents from various socioeconomic backgrounds within Oujda and neighboring areas. This allowed for the recruitment of a diverse and representative sample reflective of the local population. The objective was to assess the prevalence of eating behavior disorders - namely, anorexia nervosa, bulimia nervosa, and avoidant/restrictive food intake disorder (ARFID) - and to identify potential risk factors. Data were collected using structured questionnaires and two validated screening tools: the Eating Behavior Disorders in Youth Questionnaire (EDY-Q) and the Eating Attitudes Test (EAT-26). The mean age of participants was 14 years, with the 13-17 age group being the most affected, and the majority were female. Psychological vulnerabilities related to eating disorders were observed. Significant associations were found between susceptibility to eating disorders and factors such as older age, female gender, exposure to major family stressors, excessive media use, and academic pressure. However, socioeconomic status did not show a significant correlation. These findings suggest a notable prevalence of eating disorders among adolescents, emphasizing the urgent need for early prevention strategies and health promotion programs to mitigate long-term mental and physical health consequences.

## Introduction

Eating behavior disorders are complex and potentially life-threatening conditions characterized by disruptive eating habits that severely affect physical health and psychological well-being. These disorders encompass a broad spectrum of problematic eating behaviors, ranging from clinically diagnosable conditions such as anorexia nervosa, bulimia nervosa, and binge eating disorder to subclinical or subsyndromic variants that may still cause significant distress and impairment. Insufficient help-seeking behavior, low treatment engagement, and high dropout rates often contribute to prolonged illness, more severe outcomes, and increased healthcare costs [1].

The Diagnostic and Statistical Manual of Mental Disorders (DSM), first introduced by the American Psychiatric Association (APA) in 1952, serves as a standardized classification system for psychopathology. It provides diagnostic criteria to ensure consistent identification and treatment of mental disorders, including eating behavior disorders, across diverse clinical and research settings [2].

The etiology of eating behavior disorders is multifactorial and complex, resulting from the interaction of genetic, psychological, social, and environmental risk factors. Similar to other psychiatric conditions such as depression and anxiety, these disorders frequently emerge during vulnerable developmental periods, particularly in mid-to-late adolescence, when biological and psychosocial changes heighten susceptibility [3,4].

Understanding these disorders is crucial for child and adolescent psychologists, psychiatrists, and healthcare providers, as early identification and intervention can mitigate the risk of chronicity and adverse physical and mental health consequences. Although the focus traditionally centers on well-defined disorders such as anorexia nervosa and bulimia nervosa, many affected individuals experience subthreshold symptoms that nonetheless impact their quality of life. The subjective experience of distress combined with social dysfunction helps clinicians differentiate between normal variations in eating behavior and clinically significant pathology [5].

Given the rising prevalence and significant burden of eating behavior disorders worldwide, and the scarcity of region-specific data, particularly in Morocco, this study aims to assess the prevalence and characteristics of these disorders among children and adolescents in Oujda and its surrounding areas.

Eating disorders in this study are defined according to the DSM, Fifth Edition (DSM-5) [2], which provides standardized criteria for diagnoses such as anorexia nervosa, bulimia nervosa, and binge eating disorder. The DSM-5 criteria emphasize specific behavioral and psychological features necessary for diagnosis. While the International Classification of Diseases (ICD-10 and ICD-11) also classifies eating disorders, there are some differences in categorization and criteria. For example, ICD-11 includes avoidant/restrictive food intake disorder (ARFID) as a distinct diagnosis. Awareness of these classification systems and their nuances is important given the evolving understanding and increasing prevalence of eating disorders worldwide.

## Materials and methods

This study employed a descriptive cross-sectional design conducted over a three-month period, from January to March 2024. It was conducted at the University Hospital Center (CHU) Mohammed VI in Oujda, Morocco, and several primary and secondary educational institutions in the city. These sites were selected because of their central role in providing healthcare and education, accessibility, and their ability to represent a diverse urban population within the Oriental Region of Morocco.

The target population included children and adolescents aged 7-18 years. A total of 520 participants were randomly selected to participate in the study. Due to the absence of precise data on the total population size, a convenience sampling approach based on accessibility and voluntary participation was also used. The sample was not stratified. Inclusion criteria included age within the target range, enrollment in selected institutions, and parental consent. Participants with known psychiatric diagnoses or who refused participation were excluded. Parental consent was obtained verbally in the presence of educators or caregivers who explained the study’s objectives.

Data collection was performed using a structured, self-administered questionnaire delivered via Google Forms. Trained mediators (teachers and healthcare professionals) were present to assist participants with understanding questionnaire items if needed, ensuring consistency and reliability of responses.

Two validated screening instruments appropriate for the study population were used.

Eating Disorders in Youth Questionnaire (EDY-Q): Originally developed in German, this 14-item questionnaire includes 12 items assessing symptoms of ARFID and two additional items related to Pica and Rumination Disorder. The version used was adapted and translated into French by a panel of bilingual experts, followed by pilot testing with 20 children to ensure cultural and linguistic suitability for the Moroccan context.

Eating Attitudes Test (EAT-26): A 26-item scale widely used internationally to identify disordered eating behaviors and risk of eating disorders. The French version, validated in Francophone populations, was employed with minor adaptations after expert review to ensure relevance for Moroccan adolescents.

Both instruments were reviewed by a multidisciplinary panel of pediatricians, psychiatrists, and psychologists to verify cultural appropriateness and comprehensibility before deployment.

Data analysis was conducted using Statistical Product and Service Solutions (SPSS, version 27; IBM SPSS Statistics for Windows, Armonk, NY). Descriptive statistics summarized demographic characteristics and screening results. Associations between variables were examined using chi-square tests for categorical data and Student’s t-test for continuous variables, with statistical significance set at p < 0.05.

Finally, in this study, the term “eating disorders” is used exclusively to describe conditions meeting international diagnostic criteria as defined in the DSM-5. The broader category “eating behavior disorders” includes subclinical or emerging conditions affecting eating patterns but not fulfilling full diagnostic criteria, ensuring precision and scientific rigor in our analyses and discussions.

## Results

The sample consisted of children aged 7-18 years, with a mean age of 14 years. Among the participants, 79 were female. The 13-17 age group represented the majority of identified cases.

Regarding family structure, 91% of the children came from households with more than one child, and 50% were the eldest in their family. Analysis of body mass index (BMI) showed that 26% of the children were underweight, while 16% were classified as overweight or obese.

In terms of socioeconomic status (SES), 62% of participants belonged to the middle class. Educational level was generally average for 50% of the children, while 19% had poor academic performance.

Significant family events were reported by 47% of the children. Among them, 23% had been exposed to family conflict, 15% had divorced parents, 7% reported parental neglect, and 2% had experienced the death of a close relative.

Concerning digital media use, 58% of the children reported excessive screen exposure, including television, smartphones, and other digital devices.

In the school environment, 25% of children reported experiencing bullying, including teasing or mockery. In addition, 39% reported stress due to academic pressure, and 7% expressed feelings of social disconnection at school.

With regard to body image, nearly 50% of the children showed increased sensitivity to societal ideals of thinness and reported body dissatisfaction. Among female participants, 36% reported dissatisfaction with their body image.

The assessment of susceptibility to eating behavior disorders revealed that 22% of children exhibited signs consistent with anorexia nervosa, 13% with bulimia nervosa, 19% with pica, and 29% with ARFID. Additionally, 15% of children showed avoidant eating behaviors (Table 1).

**Table 1:** Distribution of children based on the type of eating behavior disorders to which they are susceptible, with ARFID being the most common. ARFID: Avoidant/restrictive food intake disorder

Type of eating behavior disorders	Percentage (%)
Anorexia nervosa	22%
Bulimia	13%
Pica	19%
ARFID	29%
Food Avoidance (Restrictive Eating Behaviors)	15%

Correlation analysis identified statistically significant associations between susceptibility to eating behavior disorders and several factors: age, female gender, family environment, school environment, excessive media exposure, and pubertal body changes. No significant correlation was found with SES or educational level.

Finally, Student’s t-test, with a significance level set at 5%, was used to compare groups with and without eating behavior disorders. Associations were considered statistically significant when p < 0.05 (Table 2).

**Table 2:** Statistical analysis of the association between risk factors and susceptibility to eating behavior disorders. EAT-26: Eating Attitudes Test; EDY-Q: Eating Disorders in Youth Questionnaire

Variable groups	Positive EDY-Q/EAT-26 (n=102)	Négative EDY-Q/EAT-26 (n=418)	Statistical Test	P-value
Average age	14	11.5	t(518) = 2.55	0.012
Female sex	79	184	χ²(1) = 9.30	0.002
Low socio-economic status	10	33	χ²(1) = 1.08	0.3
Poor family environment	47	79	χ²(1) = 7.50	0.007
Poor school environment	67	92	χ²(1) = 8.10	0.004
Low academic level	19	67	χ²(1) = 1.32	0.25
Excessive exposure to the media	58	87	χ²(1) = 8.90	0.003
Body changes	47	-	-	0.003

## Discussion

Eating behavior disorders can affect individuals regardless of age, gender, sexual orientation, ethnicity, or geographic location. However, adolescents and young adults are especially at heightened risk, with anorexia nervosa typically emerging earlier in life than bulimia nervosa or binge eating disorder [6].

These disorders often persist into adulthood, where their chronic and debilitating nature leads to high rates of somatic and psychiatric comorbidities, as well as significant personal and societal costs [7].

The prevalence of eating behavior disorders has risen by 25% worldwide, with approximately 10 out of every 100 adolescent females in the United States being affected [7,8]. In 2019, around 14 million individuals were affected by eating disorders, including nearly three million children and adolescents. This considerable prevalence underscores the widespread nature of eating disorders across all age groups, with a notably high proportion of those impacted being young people [9].

In Canada, pediatricians diagnosed early-onset restrictive eating behavior disorders at an incidence rate of 2.6 (95% CI: 2.1-3.2) per 100,000 person-years in children aged 5-12 [10]. The gender disparities in the incidence of eating disorders recorded in healthcare registers were striking, and eating disorders predominantly affect females [11]. It is estimated that up to 8% of women and 2% of men will experience an eating disorder at some point in their lifetime [12]. Our findings also showed that the proportion of children and adolescents with disordered eating was higher in girls than in boys, which is in line with numerous previous studies [13-15].

Most adolescents with eating behavior disorders (72.6%-88.2%) sought help for emotional or behavioral issues, primarily from mental health services, schools, general medical care, and social services [16].

The highest incidence rates for anorexia nervosa, bulimia nervosa, and eating behavior disorders not otherwise specified (EDNOS) were observed in females aged 15-19 years. In males, the highest incidence of anorexia nervosa was also observed in the 15-19-year-old age group, whereas bulimia nervosa reached its peak incidence between the ages of 20 and 29 years [17]. This increasing trend highlights the widespread nature of eating behavior disorders, making it clear that they are not confined to any specific demographic group.

According to a Finnish study, which involved a relatively small sample of 595 adolescents, a much higher incidence rate of 1,204 per 100,000 person-years, for broad anorexia nervosa was found among girls aged 15-18 [18].

However, in a cohort of Australian pediatric inpatients with newly diagnosed restrictive eating behavior disorders, only 37% met the diagnostic criteria for anorexia nervosa [19].

Our study supports previous research on body image, a key concern for adolescents. Almost half of the children in our study showed increased sensitivity to societal ideals of thinness and expressed dissatisfaction with their body image. This emphasizes the strong impact of cultural standards on young people's self-esteem and highlights the need for early intervention to promote a healthier self-image [20].

Our research found that eating behavior disorders are more prevalent among children and adolescents with higher BMI, with those who are overweight more likely to engage in disordered eating behaviors while trying to lose weight [10].

In terms of digital media consumption, many children in the study displayed excessive exposure to screens, including television, smartphones, and other digital platforms. This overexposure is linked to various negative outcomes, such as disrupted sleep patterns, increased social isolation, and the reinforcement of unrealistic body image ideals [21,22].

Media literacy programs, such as the "Media Smart" initiative in Australia and New Zealand, focus on challenging the thin ideal by encouraging critical analysis of media content. A systematic review has shown that these interventions are effective in reducing weight concerns and the internalization of media messages. The "Media Smart-Targeted" program, aimed at young women at risk, led to a 66% decrease in the development of eating behavior disorders and improved depression levels up to 12 months following the intervention [23].

## Conclusions

Eating behavior disorders constitute a significant public health issue, particularly among adolescents and young adults. Our study highlights a notable prevalence of these disorders within the studied population, confirming the urgent need for early detection and intervention. The complex, multifactorial etiology - encompassing sociocultural influences, digital media exposure, and family environment - underlines the importance of identifying key risk factors to guide effective prevention strategies.

In Morocco, despite limited epidemiological data, the rising incidence of eating behavior disorders among youth is concerning and appears linked to rapid sociocultural changes and the internalization of Western body ideals. Addressing this challenge requires enhanced training for healthcare professionals, culturally adapted prevention programs, and widespread promotion of mental health awareness and media literacy. A coordinated, multidisciplinary approach involving healthcare, educational institutions, families, and policy-makers is essential to reduce the burden of eating behavior disorders and improve adolescent health outcomes in the Moroccan context.
